# Novel β-1,4-Mannanase Belonging to a New Glycoside Hydrolase Family in Aspergillus nidulans*

**DOI:** 10.1074/jbc.M115.661645

**Published:** 2015-09-18

**Authors:** Motoyuki Shimizu, Yuhei Kaneko, Saaya Ishihara, Mai Mochizuki, Kiyota Sakai, Miyuki Yamada, Shunsuke Murata, Eriko Itoh, Tatsuya Yamamoto, Yu Sugimura, Tatsuya Hirano, Naoki Takaya, Tetsuo Kobayashi, Masashi Kato

**Affiliations:** From the ‡ Faculty of Agriculture, Meijo University, Nagoya, Aichi 468-8502, Japan,; § Graduate School of Life and Environmental Sciences, University of Tsukuba, Tsukuba, Ibaraki 305-8572, Japan, and; ¶ Graduate School of Bioagricultural Sciences, Nagoya University, Nagoya, Aichi 464-8601, Japan

**Keywords:** Aspergillus, biodegradation, fungi, glycoside hydrolase, microbiology, oligosaccharide, polysaccharide, transcription regulation

## Abstract

Many filamentous fungi produce β-mannan-degrading β-1,4-mannanases that belong to the glycoside hydrolase 5 (GH5) and GH26 families. Here we identified a novel β-1,4-mannanase (Man134A) that belongs to a new glycoside hydrolase (GH) family (GH134) in *Aspergillus nidulans*. Blast analysis of the amino acid sequence using the NCBI protein database revealed that this enzyme had no similarity to any sequences and no putative conserved domains. Protein homologs of the enzyme were distributed to limited fungal and bacterial species. Man134A released mannobiose (M_2_), mannotriose (M_3_), and mannotetraose (M_4_) but not mannopentaose (M_5_) or higher manno-oligosaccharides when galactose-free β-mannan was the substrate from the initial stage of the reaction, suggesting that Man134A preferentially reacts with β-mannan via a unique catalytic mode. Man134A had high catalytic efficiency (*k*_cat_*/K_m_*) toward mannohexaose (M_6_) compared with the *endo*-β-1,4-mannanase Man5C and notably converted M_6_ to M_2_, M_3_, and M_4_, with M_3_ being the predominant reaction product. The action of Man5C toward β-mannans was synergistic. The growth phenotype of a *Man134A* disruptant was poor when β-mannans were the sole carbon source, indicating that Man134A is involved in β-mannan degradation *in vivo*. These findings indicate a hitherto undiscovered mechanism of β-mannan degradation that is enhanced by the novel β-1,4-mannanase, Man134A, when combined with other mannanolytic enzymes including various *endo*-β-1,4-mannanases.

## Teaser

**Background:** Filamentous fungi produce various mannanolytic enzymes including β-1,4-mannanases for β-mannan degradation.

**Results:** Fungal β-1,4-mannanase, Man134A, has an unusual sequence and substrate specificity that differs from Man5C belonging to the GH5 family.

**Significance:** An *Aspergillus nidulans* β-1,4-mannanase reveals a novel glycoside hydrolase family, GH134.

## Introduction

β-Mannan polysaccharides including glucomannan, galactomannan, and galactoglucomannan are widely distributed in nature. They constitute the main components of plant cell walls and serve as storage polysaccharides in some plants. Ivory nuts, konjac, coffee beans, and red algae contain abundant amounts of β-mannan (1–4). Glucomannan consists of a β-1,4-linked backbone containing mannose or a combination of glucose and mannose moieties, and it is acetylated at the O-2 and/or O-3 positions (5, 6). Galactomannan and galactoglucomannan have branched galactose side chains linked to backbone mannoses by an α-1,6-bond (7). Konjac glucomannan and guar gum galactomannan are storage polysaccharides that are useful to the food industry because of their gelification properties (8, 9).

Mannanolytic enzymes have recently become important natural resources for industrial biorefinery processes to produce second-generation biofuels from plant biomass (10). As β-mannans have a complex structure, a set of mannanolytic enzymes with different substrate specificities is necessary for complete degradation. Filamentous fungi produce various mannanolytic enzymes including β-1,4-mannanase (EC 3.2.1.78), α-galactosidase (EC 3.2.1.22), β-mannosidase (EC 3.2.1.25), acetylmannan esterase (EC 3.1.1.6), and β-glucosidase (EC 3.2.1.21), making these organisms excellent sources of these enzymes (7, 11, 12). *Endo*-β-1,4-Mannanases that randomly hydrolyze the internal β-1,4-linkage of the mannan backbone (7) are ubiquitous in viruses, bacteria, and eukaryotes (13). They are classified according to sequence similarity into the glycoside hydrolase (GH)^2^ families GH5, GH26, and GH113 in the Carbohydrate-Active enZYmes (CAZy) database, and filamentous fungi produce β-1,4-mannanases of the GH5 and GH26 families (14).

*Aspergillus nidulans* is a classical model eukaryote for genetic and biological studies, and it produces β-1,4-mannanases and other glycoside hydrolases (12, 14). Fifteen and three genes belonging to the GH5 and GH26 families, respectively, have been identified in the *A. nidulans* genome based on the CAZy database. Among them, secreted proteins encoded by ANID_03297, ANID_03358, and ANID_06427 have known β-1,4-mannanase activity (15–18).

The production of mannanolytic enzymes is generally induced in the presence of β-mannans and regulated by the transcription factor ManR in *Aspergillus oryzae* (19, 20). Growth on konjac glucomannan is significantly decreased by disrupting *manR*. ManR-regulated genes not only include orthologs and homologs of extant glycoside hydrolase genes but also hypothetical protein genes. These findings imply that β-mannan-induced and/or ManR-regulated hypothetical genes are involved in β-mannan degradation.

Here, we investigated the enzymatic functions of a secreted hypothetical protein of which the production was induced by β-mannans. The protein shared no homology to extant β-mannanases but displayed hydrolytic activity toward β-mannan. Furthermore, the protein had no homology to any proteins with known functions, and thus we propose that the protein is a novel β-1,4-mannanase belonging to a new GH family. The β-1,4-mannanase reacted with β-mannan and manno-oligosaccharides with mannotriose recognition and had high catalytic efficiency (*k*_cat_*/K_m_*) toward mannohexaose (M_6_) compared with the *endo*-β-1,4-mannanase Man5C belonging to a GH5 family, indicating that the enzyme had a unique catalytic property. Moreover, the novel β-1,4-mannanase had a synergistic effect with Man5C toward glucomannan and galactomannan, suggesting that would be useful for diverse industrial applications including conversion technology of lignocellulosic biomass. We also show that the novel β-1,4-mannanase plays a critical role in β-mannan degradation *in vivo*.

## Experimental Procedures

### 

#### 

##### Chemicals

Mannobiose (M_2_), mannotriose (M_3_), mannotetraose (M_4_), mannopentaose (M_5_), mannohexaose (M_6_), glucomannan, galactomannan, and galactose-free β-mannan (prepared from carob galactomannan with removal of all α-galactosyl residues by α-galactosidase) were purchased from Megazyme International (Bray, Ireland). Microcrystalline cellulose (MCC; Funakoshi, Tokyo, Japan), carboxymethylcellulose (CMC; Hercules Inc., Wilmington, DE), xylan from beech wood (Sigma), and chitin (Wako Pure Chemical Industries, Osaka, Japan) served as the carbon sources in cultures and in enzymatic assays.

##### Strains, Cultures, and Media

*A. nidulans* strain A26 (*biA1*) and ABPU1 (*biA1*, *pyrG89*; *wA3*, *argB2*; *pyroA4*) were obtained from the Fungal Genetic Stock Center (Kansas State University, Manhattan, KS) and cultured on MM agar medium (10 mm NaNO_3_, 10 mm KH_2_PO_4_, 7 mm KCl, 2 mm MgSO_4_, 2 ml/liter Hutner's trace metals, 1.5% agar (pH 6.5)) containing 0.01–1.0% glucose, mannose, xylan, chitin, MCC, CMC, β-glucomannan, or β-galactomannan as the sole carbon source at 37 °C. Conidia (1 × 10^8^) of *A. nidulans* and *A. oryzae* RIB40 obtained from NITE Biological Resource Center were transferred to 300-ml flasks containing 30 ml of MM medium with 1.0% glucomannan (w/v) and rotary shaken at 120 rpm at 37 °C. Arginine (0.2 mg liter^−1^), pyridoxine (0.1 mg liter^−1^), uracil and uridine (1.12 and 1.2 g liter^−1^), and biotin (0.25 mg liter^−1^) were added to the culture medium for auxotrophic mutants.

##### Protein Identification

Mycelia were cultured in MM media containing several carbon sources for 24 h at 37 °C. Extracellular proteins from culture filtrates were separated by SDS-PAGE and two-dimensional gel electrophoresis and then stained with Coomassie Brilliant Blue. Protein bands and spots were excised from the gels, digested with trypsin, and analyzed using MALDI-TOF/TOF-MS as described (21, 22). Peptide mass fingerprints and MS/MS spectra were analyzed using theMASCOT search engine (Matrix Science Ltd., London, UK) as described (21, 22).

##### Quantitative PCR

Single-strand cDNA was synthesized from total RNA extracted from disrupted fungal cells, and then quantitative PCR proceeded as described (23). Table 1 shows the gene-specific primers. The expression of each gene was normalized against that of the actin gene (*actA*). Results are shown as relative expression.

**TABLE 1:** **Oligonucleotide primers used in this study** Gene ID numbers are according to the Aspergillus Comparative Database. Restriction sites are underlined.

Primers	Gene	Nucleotide sequence	Application
**Real time PCR**			
RTan2710-f	*AN2710*	5′-GGAAACACCCTTGATCTTGC-3′	
RTan2710-r		5′-AGGTCTTCCCATCACCGTAG-3′	
RTan6427-f	*AN6427*	5′-ACAGTGAACCTGGGAGGACA-3′	
RTan6427-r	(*Man5C*)	5′-TAACGGGTGACCATTTCCTT-3′	
RTan9276-f	*AN9276*	5′-GAGGGAATGACTGGTCTGGA-3′	
RTan9276-r		5′-TTGTCGTCGTTGTCGTTGTT-3′	
RTan2709-f	*AN2709*	5′-GCGGAATGAATGCCTACGTT-3′	
RTan2709-r		5′-TGCCTCCATCAAGACCCATT-3′	
RTao0445-f	*AO090038000445*	5′-CGGAAAAGCTCGGATGTCTA-3′	
RTao0445-r		5′-ATGGACGGCAGACTTGTAGG-3′	

**Construction of gene disruption cassette**			
an2710–5-f	*AN2710*	5′-CCTATTCCAGATTATTGCATTCAAGTT-3′	5′ region of *AN2710*
an2710–5-r		5′-AAAACCGCGAAATAAAGCTTACTGTTGACTTAAATCTGAATTG-3′	
argB-f		5′-CAATTCAGATTTAAGTCAACAGTAAGCTTTATTTCGCGGTTTT-3′	*argB*
argB-r		5′-CTCCGACTACACCCAAGCTGTCGACCTACAGCCATTGCG-3′	
an2710–3-f		5′-GCAATGGCTGTAGGTCGACAGCTTGGGTGTAGTCGGAGAG-3′	3′ region of *AN2710*
an2710–3-r		5′-CCCCGCCAGGATTCTTAACGCCC-3′	

**Plasmid for expressing ANID_02710 in *A. nidulans***			
an2710RE-f	*AN2710*	5′-GCTCTAGACCTATTCCAGATTATTGCATT-3′	pAN2710
an2710RE-r		5′-TTTTCCTTTTGCGGCCGCATATCTGATAGCTCGATGCGATTA-3′	

**Plasmids for recombinant protein production**			
an2710-f	*AN2710*	5′-CCCAAGCTTCGGCCCCCACGACGGACATGACCA-3′	pETAN2710
an2710-r		5′-CCGCTCGAGGATAGCCTGGACATCAACCCAAAAGCG-3′	
Man5C-f	*AN6427* (*Man5C*)	5′-CGGGGTACCCGCAAGGGCTTTGTGACCACCAAAGGCGA-3′	pPICZMan5C
Man5C-r		5′-ATAGTTTAGCGGCCGCCTACCGTCTCCGGTTCAACTTGTT-3′	

##### Gene Disruption of ANID_02710

Table 1 lists the primers used for gene disruption. PrimeSTAR HS DNA polymerase (Takara Bio, Otsu, Japan) was used for PCR. The 5′- and 3′-untranslated regions (1 kb) of the *ANID_02710* gene (*ANID* is the prefix of gene identifiers in the *Aspergillus* Comparative Database at the Broad Institute) were amplified by PCR using *A. nidulans* DNA and the respective primer pairs (Table 1). The *argB* gene was amplified using *A. nidulans* A26 genomic DNA and *argB-*f and *argB-*r primers. The three DNA fragments were fused by PCR using primers an2710–5-f and an2710–3-r to generate an *ANID_02710* disruption cassette that was introduced into the ABPU1 strain as described (23, 24) to create the ANID_02710 deletion strain ANID_02710Δ. Targeted gene disruption was confirmed by Southern blotting fungal total DNA using a DIG DNA labeling and detection kit (Roche Diagnostics) according to the manufacturer's instructions.

##### Introducing ANID_02710 into Gene Disruptant

A genomic DNA fragment encoding the *ANID_02710* gene with additional NotI recognition sequences was amplified using primers shown in Table 1. The DNA fragments were digested with NotI and ligated with pBS*pyrG* (23) that had been spliced with the same restriction enzyme to generate pAN2710, which was then introduced into strain ANID_02710Δ.

##### Preparation of Recombinant Proteins

We prepared *ANID_02710* cDNA by PCR using the *A. nidulans* cDNA and a set of oligonucleotide primers (Table 1). The PCR product was purified, digested by HindIII and XhoI, and then ligated to pET28a (Novagen, Darmstadt, Germany) that had been digested with the same restriction enzymes to generate pETAN2710. The pETAN2710 was introduced into *Escherichia coli* BL21-CodonPlus(DE3) (Novagen) and cultured in LB containing 50 μg ml^−1^ kanamycin sulfate for 16 h, and then a portion (2 ml) was agitated at 120 rpm in 200 ml of LB containing 50 μg ml^−1^ kanamycin sulfate at 28 °C. After the optical density reached 1.0, isopropyl-thio-β-d-galactoside (0.2 mm) was added to the medium, and the flasks were further shaken at 120 rpm for 12 h at 28 °C. The *E. coli* cells were harvested, suspended in 50 ml of 20 mm Tris-HCl buffer (pH 8.0) containing 150 mm NaCl, and disrupted by sonication. Cell-free extract was obtained from the suspension after centrifugation at 6,000 × *g* for 15 min, and then soluble fractions were separated by further centrifugation at 100,000 × *g* for 30 min. These fractions were passed through a column (φ5 × 20 mm) containing nickel-nitrilotriacetic acid-agarose (QIAgen, Hilden, Germany). The column was washed with 10 ml of 20 mm Tris-HCl buffer (pH 8.0) containing 50 mm imidazole, and proteins were eluted with the same buffer containing 300 mm imidazole. After dialysis with 20 mm Tris-HCl (pH 8.0), the protein solution was fractionated on a HiTrap Q column (1 ml, GE Healthcare) using a linear gradient of 0–0.5 m NaCl in 20 mm Tris-HCl (pH 8.0). The purified recombinant protein was finally dialyzed against 20 mm Tris-HCl (pH 8.0). All protein purification steps proceeded at 4 °C.

A cDNA fragment encoding the *Man5C* gene (*manC*; ANID_06427) was digested with KpnI and NotI and ligated into pPICZα-A (Invitrogen) that had been digested with the same restriction enzymes to generate pMan5C. The plasmid was introduced into *Pichia pastoris* KM71H (Invitrogen), and the resulting strain was cultured to produce recombinant Man5C as described (17, 18). The culture supernatant was concentrated using an Amicon Ultra filter unit (Merck-Millipore). The concentrated protein solution was fractionated on a HiTrap Q column (GE Healthcare) using a linear gradient of 0–0.5 m NaCl in 50 mm Tris-HCl (pH 8.0). The sample was then digested with endoglycosidase H (New England Biolabs, Ipswich, MA) according to the manufacturer's instructions to remove *N*-linked glycans. The deglycosylated solution was applied to a Superose 6 10/300 GL column (GE Healthcare), and recombinant proteins were eluted with 20 mm Tris-HCl (pH 8.0) containing 150 mm NaCl and dialyzed against 20 mm Tris-HCl (pH 8.0). All protein purification steps proceeded at 4 °C. Protein concentrations were assayed using Bradford Protein Assays (Bio-Rad) with bovine serum albumin as the standard.

##### Purification of Proteins from Culture Filtrate

*A. nidulans* was cultured in 2 liters of MM medium with 1.0% glucomannan as the sole carbon source for 36 h. The culture filtrate was applied to a column containing DEAE-cellulose (GE Healthcare) and equilibrated with buffer A (50 mm Tris-HCl, pH 8.0). Proteins were eluted from the column using a linear gradient of NaCl (0–0.5 m) in buffer A. Fractions containing Man5C or ANID_02710 were pooled, dialyzed against buffer A, applied to a Hi-trap Q column (GE Healthcare), equilibrated with buffer A, and then eluted with a linear gradient of NaCl (0–0.5 m) in buffer A at a flow rate of 1.0 ml min^−1^. Fractions containing Man5C or ANID_02710 product were applied to a Superose 6 10/300 GL column (GE Healthcare) equilibrated with buffer A containing 150 mm NaCl and eluted at a flow rate of 0.5 ml min^−1^. All of these steps proceeded at 4 °C. Fractions containing Man5C or ANID_02710 product served as purified preparations.

##### Enzyme Assays

β-1,4-Mannanase activity was assayed in 0.5-ml reaction mixtures containing 50 mm sodium phosphate (pH 6.0), 0.2–5% (w/v) substrates, and purified proteins or culture supernatants. Reactions were incubated at 37 °C and then stopped by boiling at 100 °C for 10 min. The reducing sugars produced by β-1,4-mannanase were measured using tetrazolium blue as described (25, 26). Standard curves were prepared based on solutions containing different concentrations of mannose. One unit of β-1,4-mannanase activity was defined as the amount of enzyme required to produce 1 μmol of reducing sugar (mannose equivalents) per min. The effect of temperature on the activity was determined using 1.0% (w/v) glucomannan as a substrate in 50 mm sodium phosphate (pH 6.0). The optimum temperature was determined by measuring the activity over the range of 30–70 °C for 15 min. The optimum pH was determined using 50 mm sodium acetate (pH 3.0–6.0), 50 mm sodium phosphate (pH 5.0–7.0), and 50 mm Tris-HCl (pH 7.0–10.0) and assayed over a range of 3.0–10.0 at 37 °C for 15 min.

The reaction products released from glucomannan, galactose-free β-mannan and manno-oligosaccharides were separated on TLC Silica gel 60 plates (Merck-Millipore) using *n*-butanol:ethanol:water (10:8:7), visualized by staining with 0.82% (v/v) *N*-(1-naphthyl)ethylenediamine dihydrochloride and 8.6% (v/v) sulfinic acid in ethanol, and baked at 105 °C for 5 min. The soluble products released from galactose-free β-mannan and manno-oligosaccharides were determined by monitoring post-column derivatized reducing sugars that were separated using a Prominence reducing-sugar HPLC analytical system (Shimadzu, Kyoto, Japan) equipped with a fluorescence detector. The supernatant was separated on a Shim-pack ISA-07/S2504 column (4.0 × 250 mm, Shimadzu, Kyoto, Japan) with a linear gradient of 0.1 m potassium borate buffer (pH 8.0) and 0.4 m potassium borate buffer (pH 9.0) for 90 min at a flow rate of 0.6 ml min^−1^. The amount of each product was quantified using manno-oligosaccharides with degree-of-polymerization (DP) values of 1 to 6 as standards. Initial rates of hydrolysis of manno-oligosaccharides by β-1,4-mannanases were determined by HPLC as decreases in substrates. The kinetic parameters *K_m_* and *k*_cat_ were calculated by fitting the Michaelis-Menten equation to initial rates using Origin Version 6.0 software (OriginLab, Northampton, MA).

##### Other Methods

The hydrolysis products of galactose-free β-mannan were analyzed using MALDI-TOF-MS as described (18). Amino acid sequences were aligned using ClustalW (27). Dry mycelial weight was determined as described (28). Phylogenetic analyses of full-length amino acid sequences proceeded by adapting the neighbor-joining method using MEGA 6 software (29).

## Results

### 

#### 

##### Identification of Extracellular Proteins Induced by β-Mannans

**FIGURE 1.**
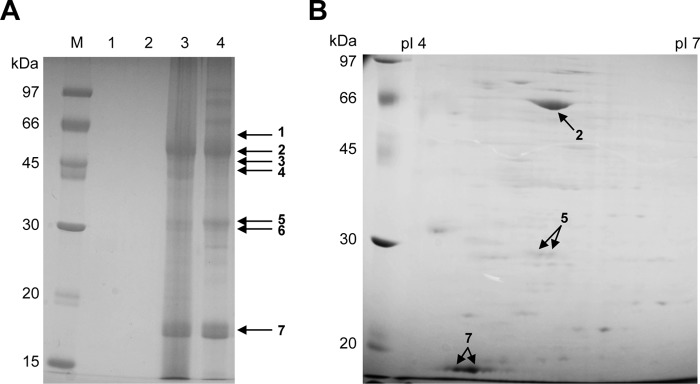
**Secreted proteins of *A. nidulans* grown with sugars and β-mannans as sole carbon source.** *A*, SDS-PAGE analysis of extracellular proteins from *A. nidulans* grown with glucose (*lane 1*), mannose (*lane 2*), galactomannan (*lane 3*), or glucomannan (*lane 4*) as the sole carbon sources for 24 h. Culture filtrates (2.5 ml) were concentrated to 25 μl using Amicon Ultra filter units and then analyzed by SDS-PAGE. Protein concentrations in culture filtrates were 10.1 and 9.3 μg/ml with galactomannan and glucomannan, respectively, as the carbon source. Proteins were not detected with glucose and mannose. *B*, expression profiles of extracellular proteins of *A. nidulans* grown in glucomannan as a sole carbon source and resolved on two-dimensional gel electrophoresis gels. About 100 μg of crude proteins of *A. nidulans* grown in MM medium containing glucomannan as the sole carbon source were resolved by two-dimensional gel electrophoresis as described under “Experimental Procedures.” *Lane M*, protein standard. *pI,* isoelectric point. *Arrows*, proteins analyzed by MALDI-TOF/TOF-MS. Identified protein bands are marked by *arrows with numbers* (Table 2).

The extracellular proteins of *A. nidulans* produced using various sole carbon sources were analyzed by SDS-PAGE (Fig. 1*A*). Whereas proteins were undetectable with glucose and mannose, three major bands (Fig. 1*A*, *arrows 2*, *5*, and *7*) comprising several proteins were detected with galactomannan and glucomannan, and seven of them were identified by peptide mass fingerprinting and MS/MS spectrum analysis using MALDI-TOF/TOF-MS (Fig. 1*A* and Table 2). The three major proteins were *endo*-β-1,4-mannanase belonging to the GH5 family (no. 2; Man5C, ANID_06427), a protease (no. 5; ANID_02366), and a hypothetical protein (no. 7; ANID_02710) (see Table 2). The others included two more GH5 family *endo*-β-1,4-mannanases (nos. 3 and 4; ANID_07639 and ANID_09276). All of them possessed signal peptides based on prediction by the downloadable SignalP server 4.0 (Technical University of Denmark, Lyngby, Denmark). The extracellular proteins of *A. nidulans* grown in MM medium containing glucomannan as the sole carbon source were also resolved by two-dimensional gel electrophoresis (Fig. 1*B*), which also identified the same proteins as those found by SDS-PAGE. These results indicated that *A. nidulans* grown with glucomannan predominantly secreted *endo*-β-1,4-mannanase (no. 2; ANID_06427) and the hypothetical protein (no. 7; ANID_02710).

**TABLE 2:** **Proteins identified in the secretome of *A. nidulans* cultured with glucomannan as sole carbon source**

No.	Annotation name	Gene ID*^a^*	Peptide mass fingerprints search			MS/MS ion search	
			Score	*M*r*^b^*	Cov.*^c^*	Score	Identified peptide sequence
1	Putative chitinase (GH18)	ANID_04871	172	44.2	44	67	IILGMPIYGR
2	*Endo*-β-1,4-Mannanase (GH5)	ANID_06427	164	45.3	34	124	LEAVGGWQSISLR, WIVDHAR, LEAVGGWQSISLR
3	Putative *endo*-β-1,4-mannanase (GH5)	ANID_07639	71	44.2	21	67	YVDSPAIFAWELANEPR
4	Putative *endo*-β-1,4-mannanase (GH5)	ANID_09276	80	41.7	22	127	TDWYTSATIQAAYR, HQGGVSTINTGQYGLQR
5	Putative trypsin-like protease	ANID_02366	84	25.4	22	72	AGYPGVYSSPAYFR
6	Putative dipeptidyl-peptidase	ANID_02572	63	79.4	13	61	FVAYAQSYR
7	Hypothetical protein	ANID_02710	136	20.9	54	88	GSYTVSGLGQR, TYDAANFGLFK

*^a^*Protein names and accession numbers are according to *Aspergillus nidulans* Genome Database.

*^b^*Theoretical mass.

*^c^*Sequence coverage (%) in peptide mass fingerprints.

##### Regulation of the Genes Encoding the Proteins Produced in the Glucomannan and Galactomannan Media

**FIGURE 2.**
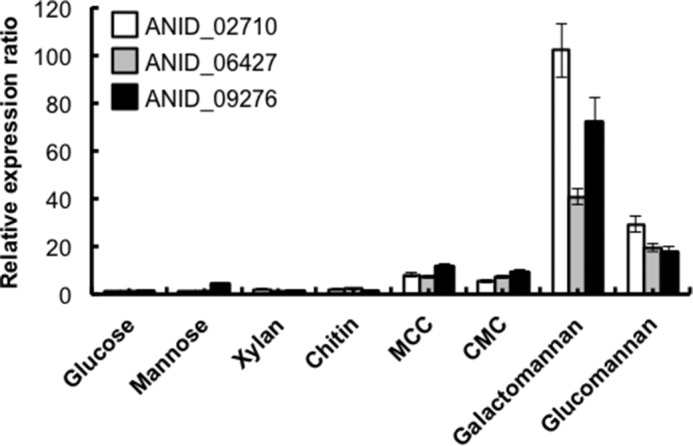
**Expression of glucomannan-induced genes identified by SDS-PAGE.** Transcripts were quantified by real-time PCR using total RNA prepared from *A. nidulans* cultured at 37 °C for 16 h. Glucose, mannose, xylan, chitin, MCC, CMC, galactomannan, or glucomannan served as sole carbon sources. Data were normalized to β-actin (*actA*) transcripts. Transcription of three genes in *A. nidulans* grown with glucose as the sole carbon source was set to 1. Data are the means of three experiments. S.D. were <9%.

Gene transcripts were quantified using real-time PCR. *A. nidulans* generated 19-, 18-, and 29-fold more *endo*-β-1,4-mannanase (*Man5C*; *ANID_06427*), putative β-mannanase (*ANID_09276*), and hypothetical protein (*ANID_02710*) transcripts, respectively, on glucomannan compared with glucose (Fig. 2). These genes were not induced in *A. nidulans* grown with mannose, xylan, and chitin as the sole carbon sources (Fig. 2). Galactomannan was more effective than glucose for expression of the genes reaching 41-, 72-, and 100-fold, respectively (Fig. 2). Both MCC and CMC slightly induced the expression of these genes (Fig. 2). In addition to the β-1,4-mannanases, ANID_02710 protein with a molecular mass of 18 kDa was produced in the presence of β-mannans (Fig. 1). These results indicated that β-mannans induced expression of the genes encoding ANID_02710 and *endo*-β-1,4-mannanases belonging to the GH5 family. The hypothetical protein XP_001825366 encoded by the AO090038000445 gene in *A. oryzae* was orthologous to the ANID_02710 product with 70% amino acid sequence identity (19). Expression levels of the *A. oryzae* gene were 62- and 37-fold higher on galactomannan and glucomannan as compared with glucose. The β-mannan induced expression of *ANID_02710* and its ortholog *AO090038000445*, suggesting that these proteins are involved in β-mannan degradation. Amino acid sequence analysis using the NCBI protein database did not reveal any putative conserved domains in the hypothetical protein.

The *ANID_02709* gene encoding a putative β-1,4-mannanase adjoins *ANID_02710*, but a transcript encoding *ANID_02709* was undetectable (data not shown), implying that ANID_02709 is not involved in β-mannan degradation. These findings also indicated that *ANID_02709* and *ANID_02710* gene expression is differently regulated in the presence of β-mannans.

##### ANID_02710 Is Involved in β-Mannan Utilization

**FIGURE 3.**
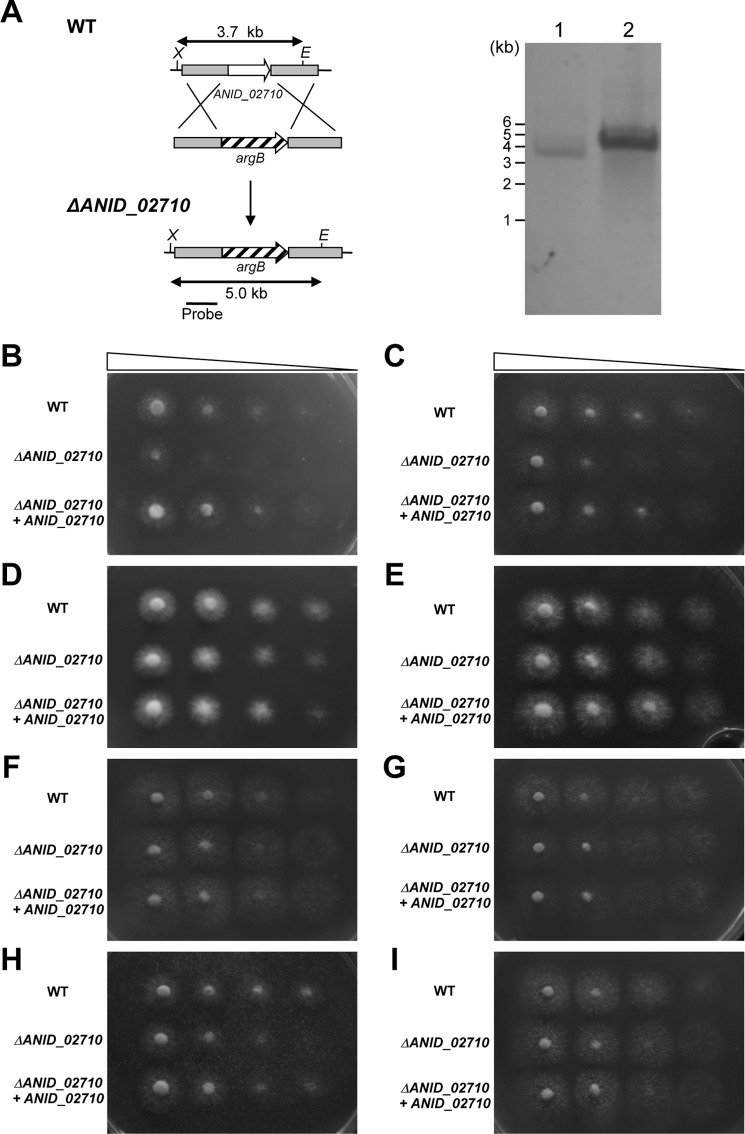
**Growth of *A. nidulans* strains on agar containing 0.05% sugars or various polysaccharides as sole carbon sources.** *A*, Disruption of *ANID_02710* gene in *A. nidulans* and introduction of *ANID_02710* allele to *A. nidulans*. Strategy for homologous recombination into *ANID_02710* locus to construct *ANID_02710* gene disruptant is shown. Total DNA from strains was digested with XhoI (*X*) and EcoRI (*E*) before Southern blotting is shown. The *bar* indicates the position and size of hybridization probe. *Lanes 1*, *A. nidulans* WT; *lane 2*, ANID_02710Δ. *B–I*: glucomannan (*B*), galactomannan (*C*), glucose (*D*), mannose (*E*), xylan (*F*), chitin (*G*), MCC (*H*), or CMC (*I*) served as carbon sources. Conidia (1 × 10^5^, 1 × 10^4^, 1 × 10^3^, 1 × 10^2^ in 5 μl) of *A. nidulans* strains were spotted onto agar plates supplemented with 0.05% sugars or polysaccharides and cultured at 37 °C for 2 days. Δ*ANID_02710*, ANID_02710Δ, Δ*ANID_02710* + *ANID_02710*, ANID_02710Δ strains were transformed into plasmid pAN2710.

We constructed a disruptant of the gene that expresses ANID_02710. We prepared an *ANID_02710* gene disruption cassette that is designed to double-crossover with the fungal chromosome at the 5′-and 3′-regions of *ANID_02710* and then introduced it into *A. nidulans*. Southern blotting and PCR analysis of total DNA from the transformant ANID_02710Δ revealed that *ANID_02710* was deleted from the strain (Fig. 3*A*). Compared with the wild type strain (WT), the phenotype of ANID_02710Δ growth was poor on agar containing either glucomannan or galactomannan as the sole carbon source (Fig. 3, *B* and *C*). The hyphal growth of the ANID_02710Δ and WT strain on agar containing any of glucose, mannose, xylan, chitin, MCC, or CMC as the sole carbon source did not significantly differ (Fig. 3, *D–I*). Introducing the *ANID_02710* gene into ANID_02710Δ restored growth in glucomannan and galactomannan medium, suggesting that that ANID_02710 protein plays a specific role in fungal β-mannan degradation.

##### ANID_02710 Product Has Mannanolytic Activity

**FIGURE 4.**
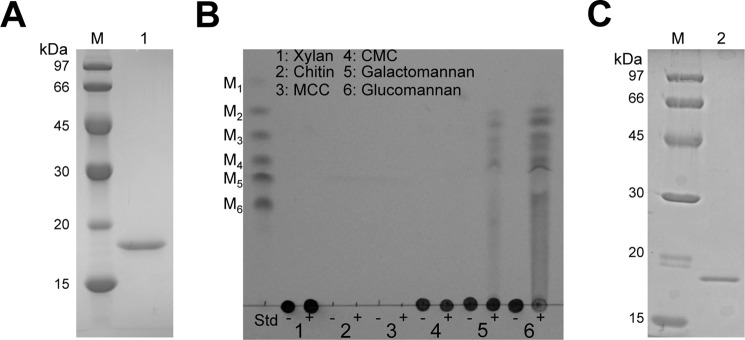
**Hydrolase activity of purified ANID_02710 protein toward various glycans.** *A*, SDS-PAGE of purified recombinant ANID_02710 product expressed in *E. coli* (*lane 1*). Purified enzyme (1 μg) was resolved by SDS-PAGE and stained with Coomassie Brilliant Blue. *Lane M*, protein standard. *B*, TLC analysis of soluble products in reaction mixtures of ANID_02710 product with various glycans. ANID_02710 protein (1.0 μm) was incubated with 0.5% substrate in 50 mm sodium phosphate buffer (pH 6.5) at 37 °C for 1 h. Products were applied, developed, and detected as described under “Experimental Procedures.” *Lane 1*, xylan; *lane 2*, chitin; *lane 3*, MCC; *lane* 4, CMC; *lane 5*, galactomannan; *lane 6*, glucomannan. Each lane included reaction mixtures with (+) and without (−) enzyme. *M_1_*, mannose; *M_2_*, mannobiose; *M_3_*, mannotriose; *M_4_*, mannotetraose; *M_5_*, mannopentaose; *M_6_*, mannohexaose; *Std*, manno-oligosaccharides standard. *C*, SDS-PAGE of purified ANID_02710 protein (0.5 μg) from *A. nidulans* (*lane 2*).

A purified recombinant ANID_02710 product was generated as an N-terminal His_6_-tagged protein in the *E. coli* expression system as described under “Experimental Procedures.” The recombinant ANID_02710 product migrated as a single band on SDS-PAGE at a molecular mass of 18 kDa after removal of the His_6_ tag by thrombin digestion followed by purification (Fig. 4*A*). The hydrolase activity of the recombinant ANID_02710 product was significant toward β-mannans but undetectable toward xylan, chitin, MCC, or CMC (Fig. 4*B*). We also purified native ANID_02710 product from culture supernatants with 40% recovery using three chromatographic separations, each of which resulted in a single band on SDS-PAGE (Fig. 4*C*). The native ANID_02710 product was essentially similar to the recombinant ANID_02710 product generated in the *E. coli* expression system (data not shown).

##### ANID_02710 Product Is a Novel Glycoside Hydrolase

**FIGURE 5.**
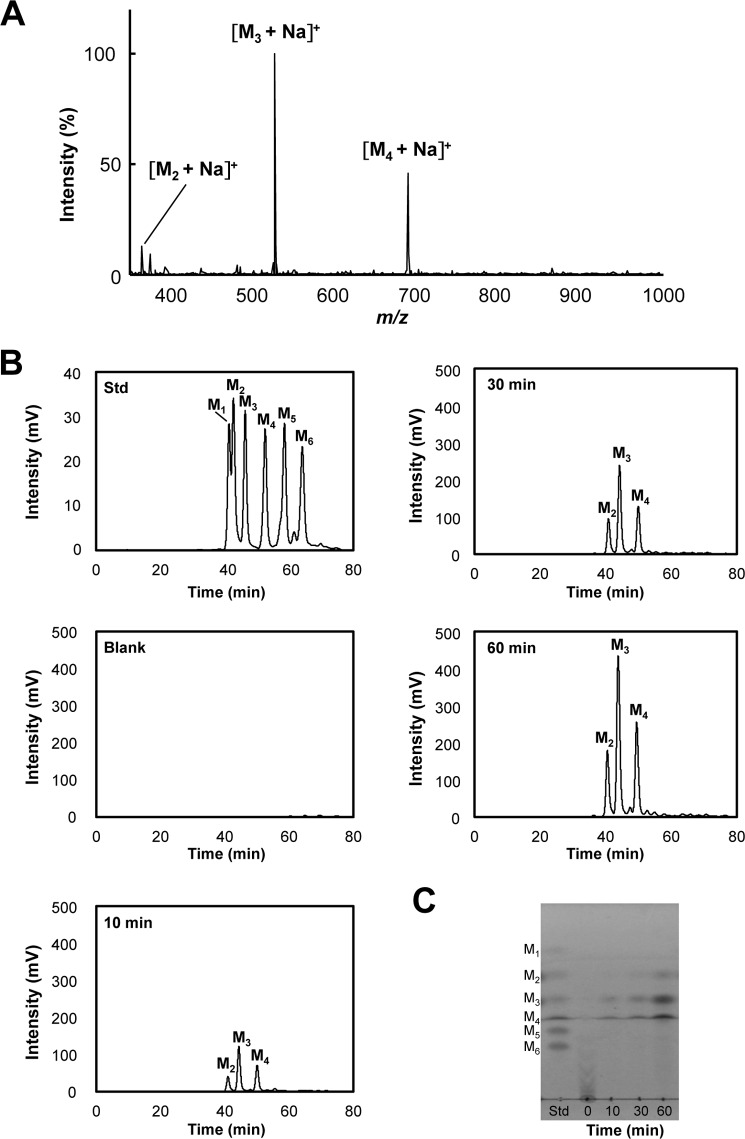
**Hydrolysis of galactose-free β-mannan by purified ANID_02710 product.** *A*, MALDI-TOF-MS analysis of hydrolytic products from galactose-free β-mannan generated by ANID_02710 product. Recombinant ANID_02710 product (1.0 μm) was incubated with 0.5% galactose-free β-mannan in 50 mm sodium phosphate buffer (pH 6.0) at 37 °C for 60 min, and then reaction products were detected as corresponding sodium adducts (molecular weight + 23). *B*, hydrolysis products of galactose-free β-mannan incubated without (*blank*) or with recombinant ANID_02710 product for 10–60 min monitored using HPLC. *C*, TLC analysis of soluble products in reaction mixtures of ANID_02710 product with galactose-free β-mannan. Recombinant ANID_02710 product (1.0 μm) was incubated with 0.5% substrate in 50 mm sodium phosphate buffer (pH 6.0) at 37 °C for 10 to 60 min. *M_1_*, mannose; *M_2_*, mannobiose; *M_3_*, mannotriose; *M_4_*, mannotetraose; *M_5_*, mannopentaose; *M_6_*, mannohexaose. *Std*, manno-oligosaccharides standard (0.5 mm each).

**FIGURE 6.**
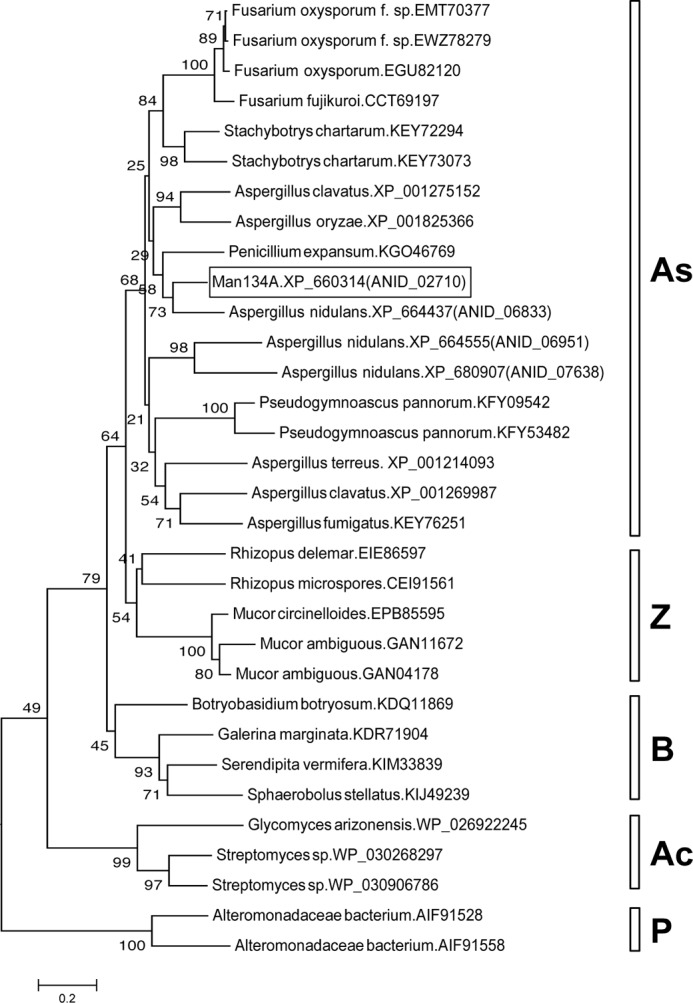
**Phylogenetic tree of GH134 family.** *Ac*, Actinobacteria; *As*, Ascomycota; *B*, Basidiomycota; *P*, Proteobacteria; *Z*, Zygomycota.

**FIGURE 7.**
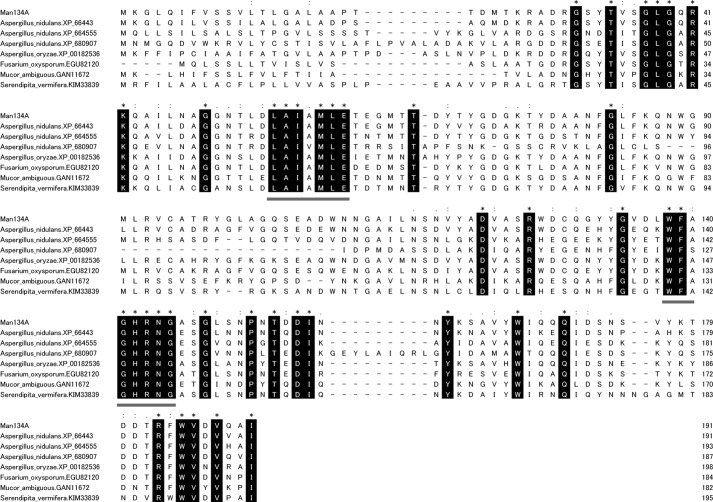
**Multiple alignment of deduced amino acid sequences of GH134 family from *A. nidulans* and its related proteins.** Amino acid sequences were aligned using ClustalW software. Identical amino acids are *highlighted. Dots and colons* indicate conserved amino acids with substitutions. *Dashes* indicate gaps. Possible sequence motifs characteristic of Man134A homologs (LAIXMLE and WFXGHRNG) are *underlined*.

The reaction products of galactose-free β-mannan generated by recombinant ANID_02710 product were analyzed using MALDI-TOF-MS (Fig. 5*A*). Considering that polysaccharides are degraded by hydrolases, lyases, and lytic polysaccharide monooxygenases, the reaction products were analyzed using MS to determine the type of reaction that the ANID_02710 product catalyzes. The molecular weights of the three products detected by MALDI-TOF-MS were identical to those of the Na^+^ adducts of mannobiose (M_2_), mannotriose (M_3_), and mannotetraose (M_4_) (Fig. 5*A*). The soluble products from galactose-free β-mannan produced by recombinant ANID_02710 product were also analyzed using HPLC and TLC (Fig. 5, *B* and *C*). Adding recombinant ANID_02710 product to the reaction mix resulted in the generation of M_2_, M_3_, and M_4_, whereas mannose (M_1_), mannopentaose (M_5_), and mannohexaose (M_6_) were undetectable (Fig. 5, *B* and *C*). These results indicated that the ANID_02710 product is a novel β-1,4-mannanase that belongs to a new family of glycoside hydrolases (family GH134 in the CAZy classification. Thus, we designated the recombinant ANID_02710 product as a β-1,4-mannanase belonging to the glycoside hydrolase 134 family (GH134). Homologs of Man134A are distributed in some Proteobacteria, Actinobacteria, Zygomycota, Basidiomycota, and Ascomycota including aspergilli except for *Aspergillus niger* (Fig. 6 and Table 3). Fig. 7 shows the sequence alignment of the Man134A orthologs. The LAIXMLE and WFXGHRNG motifs in the N-terminal and C-terminal regions, respectively, were highly conserved, suggesting that the motifs play important roles for the functions of GH134 family proteins (Fig. 7).

**TABLE 3:** **Total number of Man134A homologs in fungal genomes**

Species	Number of Man134A homologs*^a^*
**Ascomycota**	
*Aspergillus clavatus*	2
*Aspergillus fumigatus*	1
*A. nidulans*	4
*A. oryzae*	2
*A. terreus*	1
*Blastomyces dermatitidis*	
*Botrytis cinerea*	
*Candida albicans*	
*Candida guilliermondii*	
*Clavispora lusitaniae*	
*Coccidioides immitis*	
*Fusarium fujikuroi*	1
*Fusarium graminearum*	
*F.oxysporum* f. sp.	1
*F. verticillioides*	1
*Histoplasma capsulatum*	
*Lodderomyces elongisporus*	
*Neurospora crassa*	
*Microsporum canis*	
*Microsporum gypseum*	
*Penicillium expansum*	5
*Phaeosphaeria nodorum*	
*Pyrenophora tritici-repentis*	
*Schizosaccharomyces cryophilus*	
*Schizosaccharomyces japonicus*	
*Schizosaccharomyces octosporus*	
*Schizosaccharomyces pombe*	
*Sclerotinia sclerotiorum*	
*Stachybotrys chartarum*	4
*Trichoderma reesei*	
*Trichophyton equinum*	
*Trichophyton rubrum*	
*Trichophyton tonsurans*	
*Uncinocarpus reesii*	
*V. alfalfae*	1
*Verticillium dahliae*	

**Zygomycota**	
*Mucor ambiguus*	2
*Mucor circinelloides*	1
*R. delemar*	9
*Rhizopus microsporus*	5

**Basidiomycota**	
*Botryobasidium botryosum*	1
*Coprinopsis cinerea*	
*Cryptococcus neoformans*	
*Galerina marginata*	1
*Phanerochaete chrysosporium*	
*Puccinia graminis f. sp.*	
*Puccinia triticina*	
*Serendipita vermifera*	2
*Sphaerobolus stellatus*	1
*Ustilago maydis*	

*^a^*Man134A homologs include amino acid sequences having at least 50% identity to Man134A (ANID_02710).

##### Comparison of Biochemical Properties between Man134A and Man5C

**FIGURE 8.**
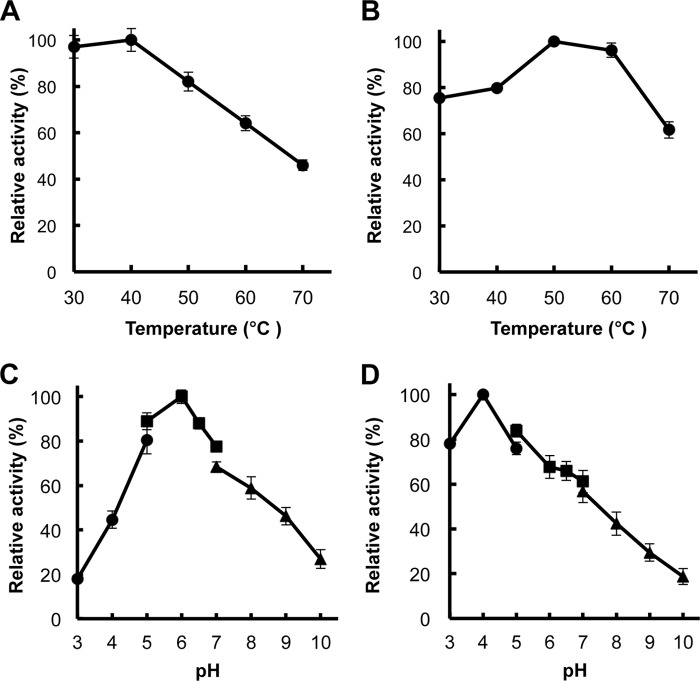
**Temperature and pH optima of rMan134A and rMan5C.** *A* and *B*, optimal temperature of rMan134A (*A*) and rMan5C (*B*). Enzyme reactions proceeded at temperatures ranging from 30 °C to 70 °C for 15 min. *C* and *D*, optimal pH of rMan134A (*C*) and rMan5C (*D*). Enzyme reactions proceeded over pH range 3.0–10.0. Buffers were 50 mm sodium acetate (pH 3.0–5.0; ●), 50 mm sodium phosphate (pH 5.0- 7.0; ■), and 50 mm glycine-NaOH (pH 7.0–10.0; ▴). *Error bars* are shown as the means ± S.E. of three independent experiments.

We compared the biochemical properties of the major mannanolytic enzymes Man134A and Man5C that were produced when glucomannan was the sole carbon source to understand how they each contribute to β-mannan hydrolysis. We prepared recombinant Man5C (rMan5C) in the *P. pastoris* expression system as described (17, 18). The optimal temperature and pH for the activities of the recombinant Man134A (rMan134A) and rMan5C were determined using glucomannan as a substrate (Fig. 8). The optimal reaction conditions for rMan134A and rMan5C were 30 °C and 50 °C, respectively (Fig. 8, *A* and *B*). The optimal pH for rMan134A activity was 6.0, and it preferred a neutral pH environment, whereas that for rMan5C activity was 4.0 (Fig. 8, *C* and *D*). These results were similar to those of native Man134A and Man5C purified from *A. nidulans* (data not shown). We investigated the kinetic parameters of Man134A and Man5C to determine the profiles of glucomannan and galactomannan hydrolysis (Table 4). The catalytic efficiency (*k*_cat_/*K_m_*) of rMan134A was 6.5-fold higher toward glucomannan (*k*_cat_/*K_m_* = 330 ml s^−1^ mg^−1^) than galactomannan (*k*_cat_/*K_m_* = 51 ml s^−1^ mg^−1^), both of which were slightly lower than those of rMan5C. Together with the activity-pH profiles in Fig. 8, these results imply that Man134A mainly contributes to β-mannan hydrolysis within a near-neutral range of pH.

**TABLE 4:** **Apparent kinetic parameters of recombinant Man134A and Man5C toward β-mannans** Data are the means ± S.D. of three experiments. Recombinant Man134A (1.0 μm) was incubated with β-mannans in 50 mm sodium phosphate buffer (pH 6.0) at 37 °C. Recombinant Man5C (1.0 μm) was incubated with β-mannans in 50 mm sodium acetate buffer (pH 4.0) at 50 °C.

Enzyme	Substrate	*K_m_*	*k*_cat_	*k*_cat_*/K_m_*
		*mg/ml*	*s*^−*1*^	*ml s*^−*1*^ *mg*^−*1*^
rMan134A	Glucomannan	1.2 ± 0.1	390 ± 30	330
	Galactomannan	4.7 ± 0.2	240 ± 20	51
rMan5C	Glucomannan	0.81 ± 0.1	540 ± 40	620
	Galactomannan	2.7 ± 0.2	200 ± 20	74

**FIGURE 9.**
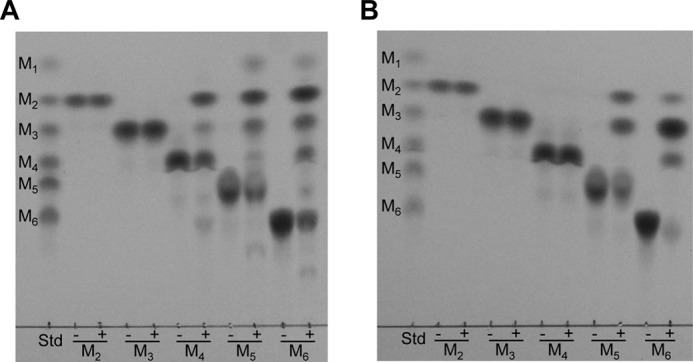
**TLC profiles of hydrolytic products generated by rMan5C and rMan134A from linear manno-oligosaccharides.** *A* and *B*, various manno-oligosaccharides (3 mm) were digested with rMan5C (*A*) and rMan134A (*B*) at 37 °C for 60 min, and reaction products were compared. Plus (+) and minus (−) indicate the presence and absence of 2.0 μm enzymes. *M_1_*, mannose; *M_2_*, mannobiose; *M_3_*, mannotriose; *M_4_*, mannotetraose; *M_5_*, mannopentaose; *M_6_*, mannohexaose; Std, manno-oligosaccharides standard (0.5 mm each).

**FIGURE 10.**
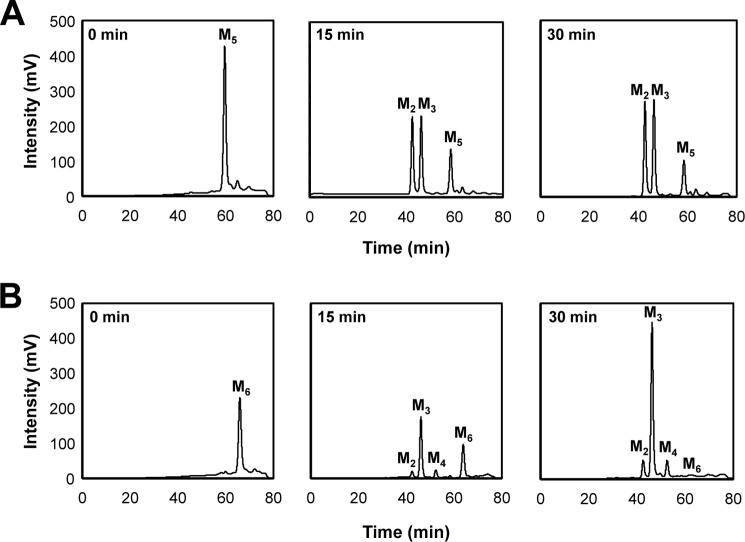
**Hydrolysis of mannopentaose and mannohexaose by rMan134A.** Reaction products of mannopentaose (*A*) and mannohexaose (*B*) catalyzed by rMan134A were sequentially monitored using HPLC. *M_1_*, mannose; *M_2_*, mannobiose; *M_3_*, mannotriose; *M_4_*, mannotetraose; *M_5_*, mannopentaose; *M_6_*, mannohexaose.

We analyzed the reaction products of linear manno-oligosaccharides generated by rMan134A and rMan5C. Recombinant Man5C hydrolyzed manno-oligosaccharides with a DP > 4 (Fig. 9*A*) and exhibited transglycosylation activity, which confirmed previous findings of recombinant Man5C expression in *P. pastoris* (17, 18). Although rMan134A did not hydrolyze M_2_, M_3_, and M_4_ (Fig. 9*B*), it produced M_2_ and M_3_ from M_5_ (Figs. 9*B* and 10*A*) and notably converted M_6_ to M_2_, M_3_, and M_4_ with M_3_ being the predominant reaction product (Figs. 9*B* and 10*B*). These findings suggested that the action of Man134A against M_6_ differs from that of other β-1,4-mannanases (26, 30–33). We also determined the transglycosylation activity of Man134A toward M_6_ in the range of 10, 20, and 30 mm together with 25, 50, and 100 mm M_4_ as a non-cleaved acceptor. However, transglycosylation activity was not detected (data not shown). These results indicated that Man134A has a unique catalytic property that hydrolyzes manno-oligosaccharides with a degree of polymerization >5, which was consistent with the results of the galactose-free β-mannan hydrolysis (Fig. 5). The substrate specificity of rMan134A toward manno-oligosaccharides was analyzed (Table 5). The catalytic efficiency (*k*_cat_/*K_m_*) of rMan134A was higher toward M_6_ than M_5_, suggesting that manno-oligosaccharides with a higher DP are preferable substrates for Man134A. The *k*_cat_/*K_m_* value of rMan134A toward M_6_ was 20-fold higher than that of rMan5C, whereas the *k*_cat_/*K_m_* values of both enzymes were similar toward M_5_ (Table 5).

**TABLE 5:** **Apparent kinetic parameters of recombinant Man134A and Man5C toward linear manno-oligosaccharides** Data are the means ± S.D. of three experiments. Recombinant Man134A (1.0 μm) was incubated with linear manno-oligosaccharides in 50 mm sodium phosphate buffer (pH 6.0) at 37 °C. Recombinant Man5C (1.0 μm) was incubated with manno-oligosaccharides in 50 mm sodium acetate buffer (pH 4.0) at 50 °C.

Enzyme	Substrate	*K_m_*	*k*_cat_	*k*_cat_*/K_m_*
		*mm*	*s*^−*1*^	*s*^−*1*^ *mm*^−*1*^
rMan134A	Mannotetraose (M_4_)			
	Mannopentaose (M_5_)	1.6 ± 0.1	76 ± 0.1	48
	Mannohexaose (M_6_)	0.083 ± 0.01	360 ± 10	4,600
rMan5C	Mannotetraose (M_4_)	3.0 ± 0.2	19 ± 2	6.3
	Mannopentaose (M_5_)	2.1 ± 0.1	120 ± 10	57
	Mannohexaose (M_6_)	0.53 ± 0.1	120 ± 10	230

##### Synergistic Action of Man134A and Man5C upon Hydrolysis of β-Mannans

**FIGURE 11.**
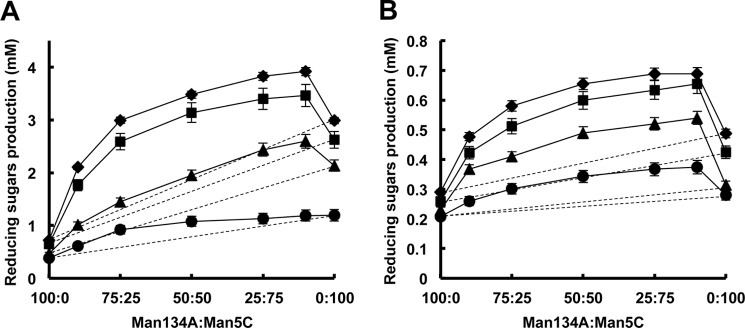
**Synergistic action of Man134A and Man5C for hydrolysis of β-mannans.** *A* and *B*, synergy plots of reducing sugar production from glucomannan (*A*) and galactomannan (*B*) by Man134A and Man5C (ratios of Man134A:Man5C of 100:0, 90:10, 75:25, 50:50, 25:75, 10:90, 0:100) after incubation for 15 min (*circles*), 30 min (*triangles*), 60 min (*squares*), and 120 min (*diamonds*). Man134A and Man5C (0.4 μm as a total) were incubated with 1.0% substrate in 50 mm sodium phosphate buffer (pH 6.0) at 37 °C. *Dashed lines* indicates the theoretical sums of reducing sugar production by the enzymes when there is no synergy.

rMan134A, rMan5C, and mixtures of the enzymes at various ratios were used for β-mannan hydrolysis to determine whether or not the enzymes have synergistic action. Fig. 11*A* shows significant increases in the production of reducing sugars from glucomannan (*solid lines*) with the enzymes at any ratio compared with the calculated sums of specific activities and amounts of enzymes (*dashed lines*). After a 120-min reaction, the production of reducing sugars reached 2.4- and 2.3-fold of the calculated values at Man134A:Man5C ratios of 10:90 and 25:75, respectively (Fig. 11*A*). Synergistic hydrolysis by the two enzymes was also evident with galactomannan as a substrate (Fig. 11*B*).

##### Man134A Is Involved in β-Mannan Degradation under Neutral pH

**FIGURE 12.**
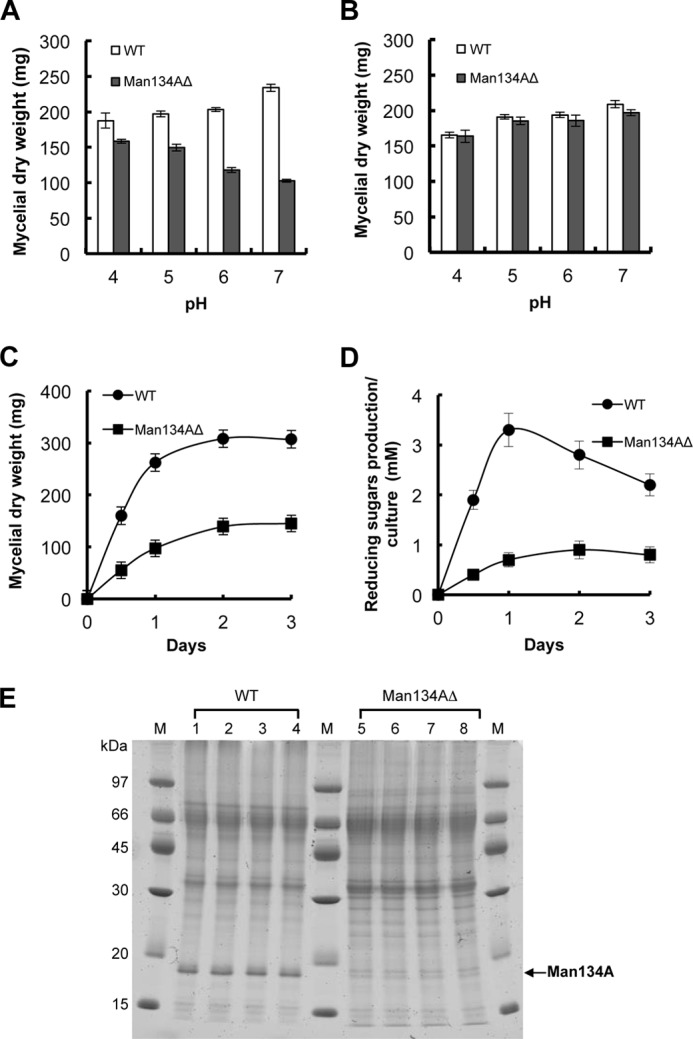
**Effects of *Man134A* gene disruption on cell growth and β-1,4-mannanase activity.** Effect of medium pH on growth of wild type and Man134AΔ strains grown in 1.0% glucomannan (*A*) or 1.0% glucose (*B*) as the sole carbon source. *A. nidulans* strains were cultured in liquid MM medium containing glucomannan or glucose as the sole carbon source at 37 °C for 24 h. Time-dependent changes in dry cell weight (*C*) and reducing sugar production by β-1,4-mannanase in culture filtrates (*D*) of *A. nidulans* strains grown 1.0% glucomannan at pH 6.5 are shown. *E*, expression profiles of extracellular proteins of WT and Man134AΔ strains. SDS-PAGE of extracellular proteins of WT (*lanes 1–4*) and Man134AΔ (*lanes 5–8*) strains grown in 1.0% glucomannan as sole carbon source. *A. nidulans* strains were cultured at pH 4.0 (*lanes 1* and *5*), 5.0 (*lanes 2* and *6*), 6.0 (*lanes 3* and *7*), and 7.0 (*lanes 4* and *8*). Crude proteins (25 μg) from 24-h cultures were separated on 12% polyacrylamide gels as described under “Experimental Procedures.”

We investigated the physiological importance of Man134A in β-mannan utilization in *A. nidulans*. Disrupting the *Man134A* gene affected fungal growth rates in liquid MM medium containing glucomannan as the sole carbon source (liquid GM medium). The growth of Man134AΔ (ANID_02710Δ was changed to Man134AΔ), and the WT strains were similar in liquid GM medium at acidic pH, whereas that of the Man134AΔ strain was defective at neutral pH (Fig. 12*A*). By contrast, glucose did not affect the hyphal growth of Man134AΔ at either acidic or neutral pH (Fig. 12*B*). We investigated the time course of the growth of Man134AΔ and the WT strains and β-1,4-mannanase activities toward glucomannan (Fig. 12, *C* and *D*). After incubation for 1 day, the dry weight of mycelia from the Man134AΔ strain was 38% that of the WT in liquid GM medium at pH 6.5 (Fig. 12*C*), and the β-1,4-mannanase activity of Man134AΔ toward glucomannan was 21% that of the WT (Fig. 12*D*). The expression of extracellular proteins other than Man134A remained essentially unchanged between WT and Man134AΔ during culture (Fig. 12*E*), indicating that Man134A is the predominant β-1,4-mannanase involved in β-mannan degradation under conditions of neutral pH in *A. nidulans*.

## Discussion

Here we identified a novel β-1,4-mannanase that shared no amino acid sequence homology with any extant β-1,4-mannanases or to any proteins with a known function and apparently lacked a conserved motif sequence. The glycoside hydrolase family GH134 was created for the enzyme in the CAZy database based on the present findings.

Filamentous fungi secrete various mannanolytic enzymes including *endo*-β-1,4-mannanases, β-mannosidases, and accessory enzymes such as α-galactosidases and acetylmannan esterases that all act in concert for efficient β-mannan hydrolysis. Expression of the mannanolytic and cellulolytic enzyme genes is controlled by the transcriptional activator ManR in response to manno-oligosaccharides as well as cellulose in *A. oryzae* (19, 20). The genes encoding the hypothetical protein XP_001825366 and β-1,4-mannanase ManG, which are orthologs of Man134A and Man5C, are also regulated by ManR in *A. oryzae*. The ortholog of ManR in *A. nidulans* is ClrB, which regulates genes encoding cellulolytic and hemicellulolytic enzymes in response to cellulose (34, 35). The ClrB target genes comprise various mannanolytic enzyme genes including the Man5C gene (*manC*). However, the Man134A gene is not included among the ClrB targets (35), suggesting that another transcription factor(s) is responsible for inducing expression of the gene. In fact, *A. oryzae* and *A. nidulans* differ in terms of regulation of the genes encoding mannanolytic enzymes. Although ManR is crucial for the β-mannan- and cellulose-induced expression of both mannanolytic and cellulolytic enzyme genes in *A. oryzae*, expression of the mannanolytic enzyme genes in *A. nidulans* is controlled by ClrB and its paralog ManS, which is not found in *A. oryzae* (36). Expression of the Man134A gene is regulated by the ClrB paralog.^3^

Our results indicated that Man134A plays an important role for growth on β-mannans at neutral pH. Some fungal β-mannanases including Man5C in this study have optimal pH for activity within the acidic range (13), whereas Man134A activity has an optimal pH near neutral. Because of this property, Man134A supports the growth of *A. nidulans* on β-mannans at neutral pH; however, no information is available about Man134A homologs in other organisms. Other features of Man134A were determined in addition to the optimal pH. One is that it released M_2_, M_3_, and M_4_ from the initial stage of reaction, with M_3_ being the predominant reaction product, when galactomannan digested with α-galactosidase was the substrate. Mannose (M_1_), M_5_, and M_6_ were not generated. This suggests exolytic hydrolysis with a preference for M_3_ units or, alternatively, endolytic initial attack followed by processive hydrolysis that releases M_3_. Transgylcosylation products were not detectable in the reaction catalyzed by Man134A. Fungal β-mannanases characterized to date belong to the GH5 and 26 families. Members of these families catalyze hydrolysis via a retaining reaction mechanism, which tends to generate transglycosylation products. These findings suggest that Man134A reacts with β-mannan via a unique catalytic mode with mannotriose recognition. The catalytic efficiency (*k*_cat_/*K_m_*) of Man134A toward M_6_ was 20-fold higher than that of Man5C, indicating that Man134A contributes to the degradation of manno-oligosaccharides, particularly those with a DP > 6. Furthermore, Man134A enhances β-mannan hydrolysis by synergistically acting with Man5C. These findings suggested that GH134 is involved in the degradation of β-mannans and especially manno-oligosaccharides with a DP > 6 released by β-1,4-mannanases.

The classification of glycoside hydrolases based on amino acid sequence similarity has led to >130 defined families being listed in the CAZy database. Families with similar three-dimensional structures have been classified into clans, and the GH5 and GH26 families that include fungal β-1,4-mannanases belong to the largest glycoside hydrolase family, GH-A. The GH-A enzymes share the triose phosphate isomerase (β/α)_8_ barrel-fold and a retaining reaction mechanism (37). The crystal structures of the β-1,4-mannanases in both GH families from a wide range of bacteria and fungi (26, 31, 33, 38, 40–42) have revealed an open active-site cleft with at least four subsites and two stringently conserved catalytic glutamates (nucleophiles and acid/base) in β-strands 4 and 7, respectively. The present study found that Man134A is the smallest protein so far identified with β-1,4-mannanase activity. Because of the absence of homology with any described functional proteins and of any putative conserved domains, the structure of Man134A and which of its amino acid residues are important for catalysis are impossible to determine without mutational and/or structural studies. The Phyre server was unable to predict the three-dimensional structure of Man134A (43). All of these imply that Man134A has a novel structure and provides insight into a novel mechanism of β-mannan hydrolysis.

Although Man134A homologs are distributed among Proteobacteria, Actinobacteria, Zygomycota, Basidiomycota, and Ascomycota (Fig. 6), only a few genera/species actually have them (Table 3). Among the bacterial genomes in the NCBI database only five encode Man134A-like proteins. Around 50 fungal genome sequences are available from the Broad Institute database, and only *A. nidulans*, *Rhizopus delemar* RA 99–880, *Fusarium oxysporum* 4287 (FO2), *Fusarium verticillioides* 7600 (FV3), *Aspergillus terreus*, and *Verticillium alfalfae* VaMs.102 encode Man134A-like proteins. Homologs have not been detected in plants and animals. The distribution of homologs suggests horizontal gene transfer including that between bacteria and fungi. Some plant pathogenic bacteria and fungi possess Man134A homologs. Although the physiological functions of the homologs remain obscure, they might be involved in the degradation of cell wall polysaccharides to infect plants.

The diverse industrial applications of β-mannanases include de-inking paper waste, clarifying fruit juices, upgrading feed, fodder, and fibers, and saccharifying plant biomass. The novel β-mannanase described herein should be of interest to the food and pulp industries and to studies of the conversion technology of lignocellulosic biomass (39).

In conclusion, the present study identified a small β-1,4-mannanase, Man134A, belonging to a new glycoside hydrolase family produced in *A. nidulans* and found that it is involved in the fungal degradation of β-mannans. Man134A had unique substrate specificity with catalytic efficiency (*k*_cat_*/K_m_*) toward M_6_ that was as high as that of Man5C and synergistically enhanced β-mannan degradation with Man5C, suggesting that GH134 acts in concert with other mannanolytic enzymes in filamentous fungi to degrade hemicellulosic biomass.

## Author Contributions

M. S., N. T., T. K., and M. K. planned the studies and prepared the manuscript. M. S., Y. K., S. I., M. M., and K. S. designed and performed the experiments. M. Y., S. M., E. I., T. Y., Y. S., and T. H. performed the experiments. All authors reviewed the results, contributed to the writing, and approved the manuscript.

## Footnotes

3 N. Li, E. Kunitake, A. Watanabe, K. Kanamaru, M. Kimura, and T. Kobayashi, unpublished results.

## Footnotes

2 The abbreviations used are:

GH: glycoside hydrolase
CAZy: Carbohydrate-Active enZYmes
CMC: carboxymethyl cellulose
DP: degree of polymerization
MCC: microcrystalline cellulose
-f: forward
-r: reverse.
